# Effects of Diphenyl Diselenide on Methylmercury Toxicity in Rats

**DOI:** 10.1155/2013/983821

**Published:** 2013-12-29

**Authors:** Cristiane L. Dalla Corte, Caroline Wagner, Jéssie H. Sudati, Bruna Comparsi, Gerlania O. Leite, Alcindo Busanello, Félix A. A. Soares, Michael Aschner, João B. T. Rocha

**Affiliations:** ^1^Biochemistry and Molecular Biology Department, Graduation Program in Biological Sciences: Toxicological Biochemistry, Natural and Exact Sciences Center, Federal University of Santa Maria, 97105-900 Santa Maria, RS, Brazil; ^2^Federal University of Pampa—Caçapava do Sul Campus, Avenida Pedro Anunciação, Vila Batista, 96570-000 Caçapava do Sul, RS, Brazil; ^3^Higher Education Cenecista Institute of Santo Ângelo—IESA, Rua Dr. João Augusto Rodrigues 471, 98801-015 Santo Ângelo, RS, Brazil; ^4^Regional University of Cariri, Pharmacology and Molecular Chemistry Laboratory, Rua Cel. Antônio Luís 1161, 63100-000 Crato, CE, Brazil; ^5^Department of Pharmacology, Vanderbilt University Medical Center, Nashville, TN 37232, USA; ^6^Department of Pediatrics, Vanderbilt University Medical Center, Nashville, TN 37232, USA

## Abstract

This study investigates the efficacy of diphenyl diselenide [(PhSe)_2_] in attenuating methylmercury- (MeHg-)induced toxicity in rats. Adult rats were treated with MeHg [5 mg/kg/day, intragastrically (i.g.)] and/ or (PhSe)_2_ [1 mg/kg/day, intraperitoneally (i.p.)] for 21 days. Body weight gain and motor deficits were evaluated prior to treatment, on treatment days 11 and 21. In addition, hepatic and cerebral mitochondrial function (reactive oxygen species (ROS) formation, total and nonprotein thiol levels, membrane potential (ΔΨm), metabolic function, and swelling), hepatic, cerebral, and muscular mercury levels, and hepatic, cerebral, and renal thioredoxin reductase (TrxR) activity were evaluated. MeHg caused hepatic and cerebral mitochondrial dysfunction and inhibited TrxR activity in liver (38,9%), brain (64,3%), and kidney (73,8%). Cotreatment with (PhSe)_2_ protected hepatic and cerebral mitochondrial thiols from depletion by MeHg but failed to completely reverse MeHg's effect on hepatic and cerebral mitochondrial dysfunction or hepatic, cerebral, and renal inhibition of TrxR activity. Additionally, the cotreatment with (PhSe)_2_ increased Hg accumulation in the liver (50,5%) and brain (49,4%) and increased the MeHg-induced motor deficits and body-weight loss. In conclusion, these results indicate that (PhSe)_2_ can increase Hg body burden as well as the neurotoxic effects induced by MeHg exposure in rats.

## 1. Introduction

MeHg is one of the most poisonous environmental contaminants, causing toxic effects in humans and experimental animals [1, 2]. Environmental MeHg is largely derived from inorganic mercury biomethylation carried out primarily by aquatic microorganisms [3] with subsequent accumulation in the aquatic food chain and human consumption [4]. MeHg causes acute and chronic damage to multiple organs, most profoundly to the central nervous system (CNS), in particular when exposures occur during the neurodevelopmental period [1, 5, 6].

The events that mediate MeHg toxicity are largely dependent upon its electrophilic properties, which allow for its interaction with soft nucleophilic groups (mainly thiols and selenols) from either low- or high-molecular-weight biomolecules [7]. The interaction of MeHg with soft nucleophilic groups from biomolecules is responsible, at least in part, for decreased antioxidant capacity and increased ROS generation [7, 8]. Notably, MeHg can disrupt the activity of thiol- and selenol-containing proteins, such as glutathione peroxidase (GPx), thioredoxin (Trx), and TrxR [1, 9–11]. These proteins are important components of the cellular antioxidant system, and their inhibition contributes to the disruption of the normal redox balance of cells [7].

In addition, MeHg can disrupt mitochondrial function by targeting specific thiol-containing proteins, including respiratory chain complexes [12, 13]. The inhibition of these complexes or enzymes can contribute to mitochondrial depolarization and swelling upon MeHg exposure. Mitochondrial targeting by MeHg has also been associated with increased mitochondrial ROS generation, which can further exacerbate the toxicity of MeHg by attacking additional nucleophilic centers in mitochondria and in other subcellular compartments [7, 10, 12–14], leading to a vicious cycle of cell demise.

Several studies demonstrated that organic and inorganic selenium (Se) compounds influence the deposition and toxicity of MeHg [13, 15, 16]. Se is an essential trace element for a wide range of living organisms, including humans [17]. Se is necessary for the expression of approximately 25 Se-dependent proteins, including GPx, TrxR, and several other enzymes and proteins, which can modulate the cellular redox and antioxidant status [17].

In addition to inorganic and naturally occurring organoselenium compounds, synthetic organoselenium compounds can also exhibit protective effects against MeHg. For example, ebselen and (PhSe)_2_ have been shown to exert beneficial effects against *in vitro* and *in vivo* MeHg-induced toxicity [18–21]. (PhSe)_2_ (which is the simplest of the diaryl diselenides [22]) protected against an array of toxic effects of MeHg and lowered the Hg burden in the brain, liver, and kidneys of adult mice [21]. The molecular mechanism(s) which underlie(s) the protective effects of (PhSe)_2_ in mice likely reflect the direct interaction of MeHg with “selenol intermediate” of (PhSe)_2_ after its reaction with thiols, or indirectly, by modulating oxidative stress levels [21, 23]. In short, the protective effects of (PhSe)_2_ against MeHg-induced toxicity are likely related to its antioxidant properties and its ability to form stable complexes with MeHg, which can increase Hg excretion and decrease the MeHg body burden.

Of particular pharmacological significance, the toxicity and pharmacokinetics of MeHg [24] are different in mice and rat which can be explained by the higher binding affinity of rat hemoglobin, which contains more cysteinyl residues than mice protein, for MeHg when compared to the mice hemoglobin [25]. (PhSe)_2_ toxicity and pharmacokinetics differences between mice and rat also exist and could be explained by a faster metabolization of (PhSe)_2_ in mice [26–28].

Therefore, the aim of the present study was to investigate the potential protective effects of (PhSe)_2_ against MeHg-induced toxicity and mitochondrial dysfunction in rats. To accomplish this goal, the effects of (PhSe)_2_ on Hg deposition in liver and brain and on behavioral and biochemical parameters were studied in rats.

## 2. Materials and Methods

### 2.1. Chemicals

Chemicals, including ethylene glycol-bis(*β*-aminoethylether)-*N,N,N*′*,N*′-tetraacetic acid (EGTA), 4-(2-hydroxyethyl)-1-piperazineethanesulfonic acid (HEPES), 2,4 dinitrophenol (2,4 DNP), 3-(4,5-dimethylthiazol-2-yl)-2,5-diphenyltetrazolium bromide (MTT), glutamic acid, safranin O, 2′,7′-dichlorofluorescin diacetate (H_2_-DCFDA), and methylmercury chloride were obtained from Sigma Aldrich (St. Louis, MO, USA). (PhSe)_2_ was synthesized according to the method by Paulmier [29]. All other chemicals were of analytical reagent grade and purchased from local commercial suppliers.

### 2.2. Animals

Male Wistar rats, weighing 250–310 g and with age from 3 to 3.5 months, from our own breeding colony were kept in cages (four animals in each). Rats were placed in a room with controlled temperature (22 ± 3°C) on a 12 h light/dark cycle (lights on at 7:00 a.m.) and had continuous access to food and water. All experiments were conducted in accordance with the Committee on Care and Use of Experimental Animal Resources of the Federal University of Santa Maria, Brazil.

### 2.3. Treatment

Sixteen rats were equally divided into four experimental groups as follows: (1) control (10 mL/Kg of water i.g. and 1 mL/Kg of soybean oil i.p.); (2) (PhSe)_2_ (10 mL/Kg of water i.g. and 1 mg/Kg of (PhSe)_2_ i.p.); (3) MeHg (5 mg/Kg of MeHg i.g. and 1 mL/Kg of soybean oil i.p.); and (4) (PhSe)_2_ + MeHg (5 mg/Kg of MeHg i.g. and 1 mg/Kg of (PhSe)_2_ i.p.). Based on previous studies, exposures were performed daily over a 21-day period [21, 30, 31]. Twenty-four hours after the last exposure, the animals were sacrificed and the livers, brains, kidneys, and skeletal muscle were quickly removed, placed on ice and homogenized.

### 2.4. Determination of Hg Levels

Tissue levels of total Hg were measured in liver, brain, and skeletal muscle collected at the time of euthanasia [32]. Approximately 0.4 g (wet weight) of the tissues was weighed and digested with 5 mL of HNO_3_ acid (65%). Digested samples were diluted to 50 mL with ultrapure water before analysis using a Multitype ICP Emission Spectrometer (ICPE-9000, Shimadzu). Calibration standard curve was prepared freshly using mercury stock standard solution.

### 2.5. Motor Coordination Tests

#### 2.5.1. Open Field Test

General locomotor activity was evaluated as previously described [33]. The number of line crossings (number of segments crossed with the four paws) and rearings was measured over 5 min and taken as an indicator of locomotor activity. The test was carried out at 3 time points: 24 hours prior to treatment (basal), and on treatment days 11 and 21.

#### 2.5.2. Rotarod Test

Motor coordination was tested on the rotarod apparatus as described previously [34, 35]. The latency to fall and the number of falls from the apparatus were recorded for 120 s. The tests were conducted 3 times: 24 hours prior to treatment (basal), and on treatment days 11 and 21.

### 2.6. TrxR

#### 2.6.1. TrxR Purification

TrxR was partially purified by a modification of the method by Holmgren and Bjornstedt [36]. Tissues were homogenized in buffered saline (137 mM NaCl, 2.7 mM KCl, 4.3 mM Na_2_HPO_4_, and 1.4 mM KH_2_PO_4_, pH 7.3). Livers, brains, and kidneys (0.5 g) were homogenized in 10, 3, and 5 volumes of buffered saline, respectively. Homogenates were centrifuged at 13,000 g for 30 min. The protein concentration in the supernatant was measured and adjusted to 10 mg/mL. The supernatant was dialyzed against buffered saline for 16 h to remove endogenous glutathione (GSH) and Trx. The dialysate was heated at 55°C for 10 min, cooled, and centrifuged at 13,000 g for 30 min to remove denatured protein.

#### 2.6.2. TrxR Activity

TrxR activity was measured by the method of Holmgren and Bjornstedt [36]. The reaction mixture consisted of the following: 0.24 mM NADPH, 10 mM EDTA, 100 mM potassium phosphate buffer (pH 7.0), 2 mg/mL 5,5′ dithiobis-2-nitrobenzoic acid (DTNB), and 0.2 mg/mL of BSA. The partially purified TrxR was added (to final concentration of 6–8 *μ*g of protein) to the cuvette containing the reaction mixture, and the absorbance was followed at 412 nm for a maximum of 4 min.

### 2.7. Isolation of Rat Brain and Liver Mitochondria

Rat brain and liver mitochondria were isolated as previously described by Brustovetsky and Dubinsky [37], with some modifications. Brain and liver were rapidly weighed and homogenized in 1 : 5 (w/v) ice-cold “isolation buffer I” containing 225 mM mannitol, 75 mM sucrose, 1 mM K^+^-EGTA, 0.1% bovine serum albumin (BSA), and 10 mM K^+^-HEPES, pH 7.2. The tissue was then manually homogenized with a potter glass. The resulting suspension was centrifuged for 7 min at 2,000 g. After centrifugation the supernatant was recentrifuged for 10 min at 12,000 g. The pellet was resuspended in “isolation buffer II” containing 225 mM mannitol, 75 mM sucrose, 1 mM K^+^-EGTA, and 10 mM K^+^-HEPES pH 7.2 and recentrifuged at 12,000 g for 10 min. The supernatant was discarded and the final pellet gently washed and resuspended in “isolation buffer II” without EGTA.

### 2.8. Mitochondrial Nonprotein and Total Thiol Content

Mitochondrial nonprotein and total thiol content were measured according to the method of Ellman [38]. To determine total thiol groups, mitochondria (0.3 mg protein) were added to the reaction medium containing 10 mM Tris-HCl pH 7.2, 1% SDS, and 10 mM DTNB. Nonprotein thiol content was measured by adding 50 *μ*L 10% TCA to 50 *μ*L of the mitochondria (0.3 mg protein). After centrifugation (4,000 ×g at 4°C for 10 min), the protein pellet was discarded and an aliquot of the clear supernatant, neutralized with 0.1 M NaOH, was added to the medium containing 10 mM Tris-HCl pH 7.2 and 10 mM DTNB. The samples absorbance was measured spectrophotometrically at a wavelength of 412 nm.

### 2.9. Measurements of Mitochondrial ΔΨm

Mitochondrial ΔΨm was estimated by fluorescence changes in safranin O (3 mM) recorded by RF-5301 Shimadzu spectrofluorometer (Kyoto, Japan) operating at excitation and emission wavelengths of 495 and 535 nm, with slit widths of 1.5 nm [39]. Data on ΔΨm in the figures is presented in Arbitrary Fluorescence Units (AFU).

### 2.10. Estimation of ROS Production

The mitochondrial generation of ROS was determined spectrofluorimetrically, using the membrane permeable fluorescent dye H_2_-DCFDA recorded by RF-5301 Shimadzu spectrofluorometer (Kyoto, Japan) operating at excitation and emission wavelengths of 488 and 525 nm, with slit widths of 3 nm [40]. Data of ROS production in the figures is presented as Arbitrary Fluorescence Units (AFU).

### 2.11. Assessment of Mitochondrial Metabolic Function

The mitochondrial metabolic function was assessed by the conversion of MTT to a dark violet formazan product by mitochondrial dehydrogenases [41]. The rate of MTT reduction was measured spectrophotometrically at a wavelength of 570 nm. Results were expressed as the percentage of MTT reduction relative to control values.

### 2.12. Assessment of Mitochondrial Swelling

Measurement of mitochondrial swelling was performed in a RF-5301 Shimadzu spectrofluorometer at 600 nm (slit 1.5 nm for excitation and emission) [42]. Data for mitochondrial swelling are expressed as Arbitrary Absorbance Units (AAU). The difference (Δ*A*) between the initial absorbance reading and the final absorbance reading was used for statistical analysis.

### 2.13. Protein Measurement

Protein was assayed by the method of Bradford [43] with bovine serum albumin as standard.

### 2.14. Statistical Analysis

Normality assumption was tested with Kolmogorov-Smirnov test and the distribution of the majority of results is not normal. Data were analyzed statistically by Mann-Whitney or Kruskal-Wallis, followed by Dunn's post-hoc tests when appropriate. The results were considered statistically significant at *P* < 0.05. All statistical analyses were conducted using GraphPad Prism 5 (Version 5.01, GraphPad Software, Inc., USA).

## 3. Results

### 3.1. Effects of (PhSe)_2_ and MeHg on Body Weight

**Figure 1:**
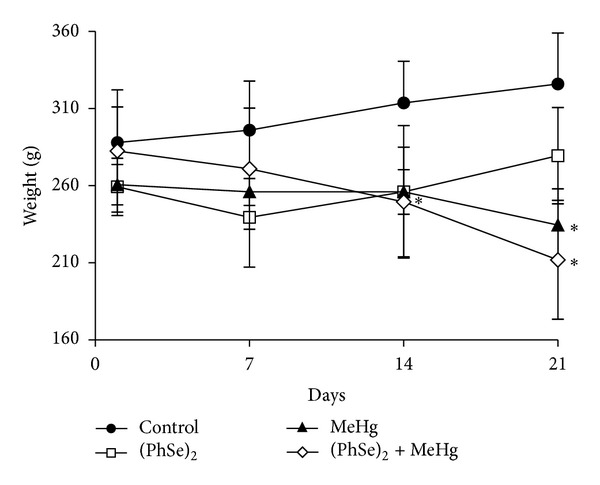
Effect of MeHg and/or (PhSe)_2_ on the body weight gain in adult rats. Data are expressed as mean ± S.D., *n* = 4. (∗) represents *P* < 0.05 as compared to controls by Mann-Whitney test.

Treatment with MeHg led to body-weight loss from the second week until the end of the treatment compared to controls (*P* < 0.05, Figure 1). Rats cotreated with (PhSe)_2_ and MeHg also showed a decrease in the body weight when compared to the control group (*P* < 0.05, Figure 1). Rats treated with (PhSe)_2_ lost weight after the first week of treatment (*P* < 0.05) but showed a trend towards a recovery and were statistically indistinguishable from the controls at the end of the treatment (Figure 1).

### 3.2. Effects of (PhSe)_2_ and MeHg on Hg Deposition

**Figure 2:**
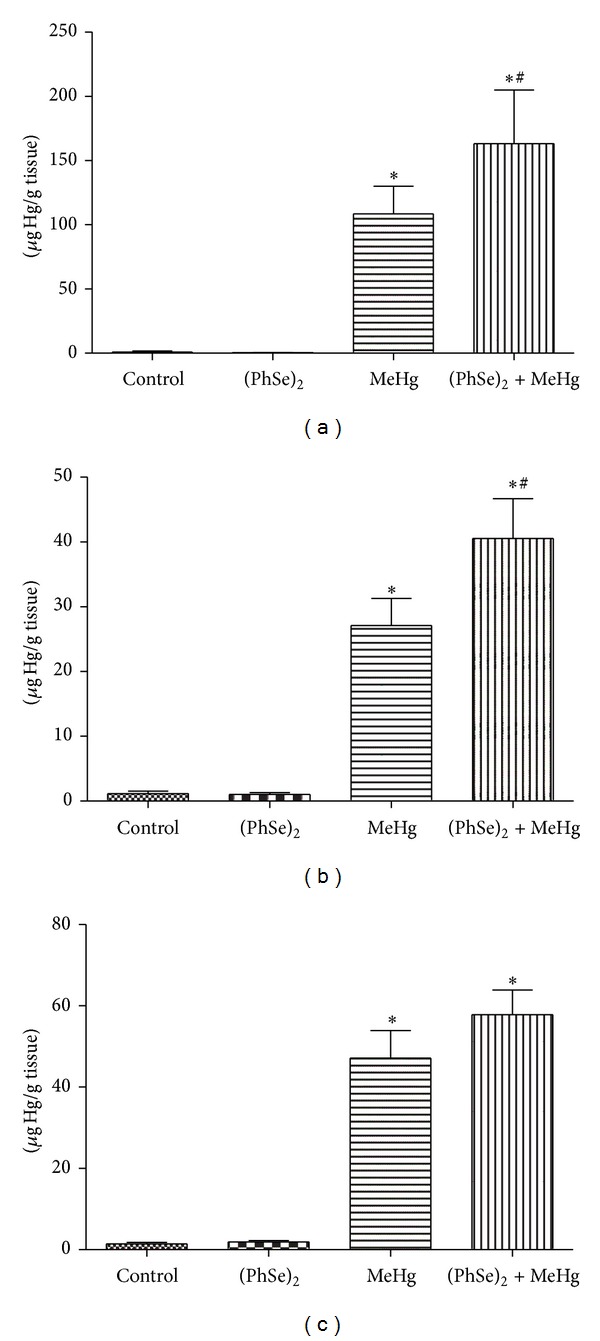
Hg content in liver (a), brain (b), and muscle (c) of rats exposed to MeHg and/or (PhSe)_2_. Data are expressed as mean ± S.D., *n* = 4. (∗) represents *P* < 0.05 as compared to controls by Mann-Whitney test. (#) represents *P* < 0.05 as compared to MeHg by Mann-Whitney test.

Treatment with MeHg increased the levels of Hg in liver, brain, and skeletal muscle compared with controls (*P* < 0.05, Figure 2). The cotreatment with (PhSe)_2_ caused a greater increase in brain Hg deposition when compared to MeHg alone treatment, both in brain (Figure 2(b)) and liver (Figure 2(a)), and showed a trend towards increased deposition in skeletal muscle (Figure 2(c)).

### 3.3. Effects of (PhSe)_2_ and MeHg on Motor Coordination and Spontaneous Locomotor Activity

**Figure 3:**
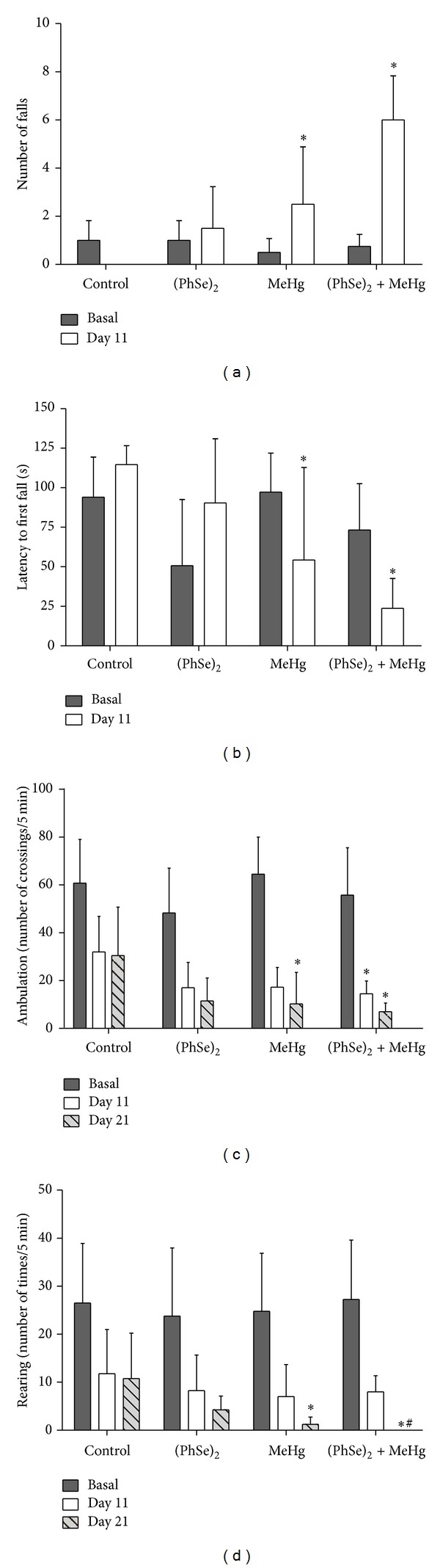
Rotarod and open field tests in rats exposed to MeHg and/or (PhSe)_2_. The number of falls (a) and latency for the first fall (b) ambulation (crossing) (a) and rearing (b) were recorded. Data are expressed as mean ± S.D., *n* = 4. (∗) represents *P* < 0.05 as compared to controls by Kruskal-Wallis test followed by multiple comparison test. (#) represents *P* < 0.05 as compared to (PhSe)_2_ by Kruskal-Wallis test followed by multiple comparison test.

The effects of MeHg and/or (PhSe)_2_ on locomotion and motor coordination were assessed by the open-field and rotarod tests, respectively. After 11 days, rats treated with MeHg showed increased number of falls on the rotarod and decreased latency to the first fall when compared to controls (*P* < 0.05, Figures 3(a) and 3(b)). Rats treated with (PhSe)_2_ did not show statistically significant differences on the rotarod test when compared to controls; however, rats cotreated with (PhSe)_2_ and MeHg showed increased loss of motor coordination as evidenced by increased number of falls and reduced latency to the first fall (*P* < 0.05, Figures 3(a) and 3(b)). The rotarod test could not be performed at the end of the treatment in rats receiving MeHg since they were unable to remain in the apparatus due to severe motor impairment caused by MeHg.

Rats treated with MeHg showed a decrease in the number of crossings and rearings in the open-field at the end of the treatment compared to the control rats (*P* < 0.05, Figures 3(c) and 3(d)). Rats cotreated with MeHg and (PhSe)_2_ also showed a significant decrease in the number of crossings after 11 days of treatment and a decrease in the number of rearings at the end of the treatment (*P* < 0.05, Figure 3(d)). Treatment with (PhSe)_2_ did not affect the rats' performance in the open-field. The decrease in the number of crossings and rearings observed in all groups on treatment days 11 and 21 was expected given that the animals habituate to the open-field arena [44].

### 3.4. Effects of (PhSe)_2_ and MeHg on Mitochondrial Dysfunction

#### 3.4.1. Mitochondrial Metabolic Function

**Figure 4:**
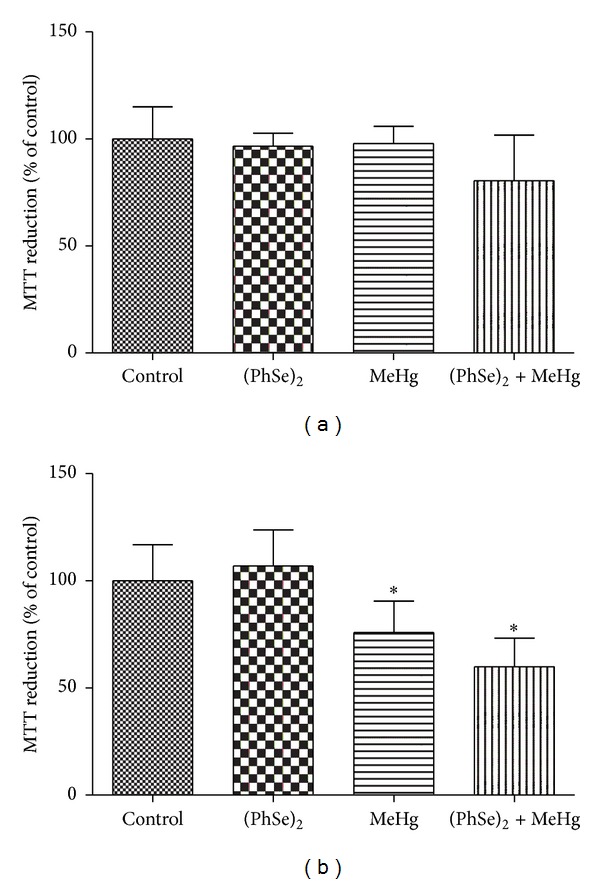
MTT reduction in liver (a) and brain (b) mitochondria of rats exposed to MeHg and/or (PhSe)_2_. Data are expressed as mean ± S.D., *n* = 4. (∗) represents *P* < 0.05 as compared to controls by Mann-Whitney test.

The hepatic mitochondrial metabolic integrity (MTT reduction) was not affected by MeHg and/or (PhSe)_2_ (Figure 4(a)). Treatment with MeHg or cotreatment with MeHg and (PhSe)_2_ decreased the capacity of brain mitochondrial dehydrogenases to reduce MTT compared to controls (*P* < 0.05, Figure 4(b)). Treatment with (PhSe)_2_ alone did not affect the cerebral mitochondrial metabolic function.

#### 3.4.2. Mitochondrial Total and Nonprotein Thiols

**Figure 5:**
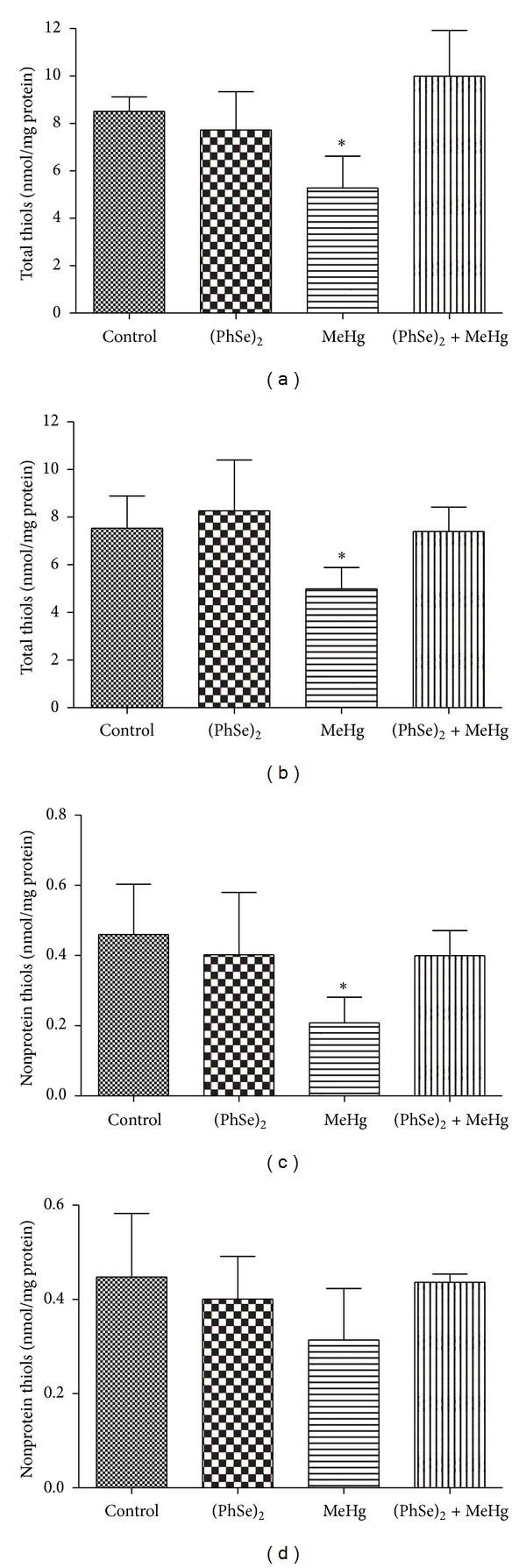
Total and nonprotein thiol content in liver (a), (c) and brain (b), (d) mitochondria of rats exposed to MeHg and/or (PhSe)_2_. Data are expressed as mean ± S.D., *n* = 4. (∗) represents *P* < 0.05 as compared to controls by Mann-Whitney test.

MeHg treatment decreased the total mitochondrial thiol levels in brain and liver when compared to controls (*P* < 0.05, Figure 5). Treatment with (PhSe)_2_ alone did not alter the mitochondrial total thiol levels in liver and brain (Figure 5). The cotreatment with (PhSe)_2_ blunted the MeHg-induced mitochondrial total thiol level depletion in rats' liver and brain (*P* < 0.05, Figure 5). Rats treated with MeHg showed decreased mitochondrial nonprotein thiol levels in the liver compared to controls, and coadministration of (PhSe)_2_ blunted the MeHg-induced decrease in hepatic nonprotein thiol content (*P* < 0.05, Figure 5(a)). Brain mitochondrial nonprotein thiol levels were not affected by any of the treatments (Figure 5(b)).

#### 3.4.3. Mitochondrial Swelling

**Figure 6:**
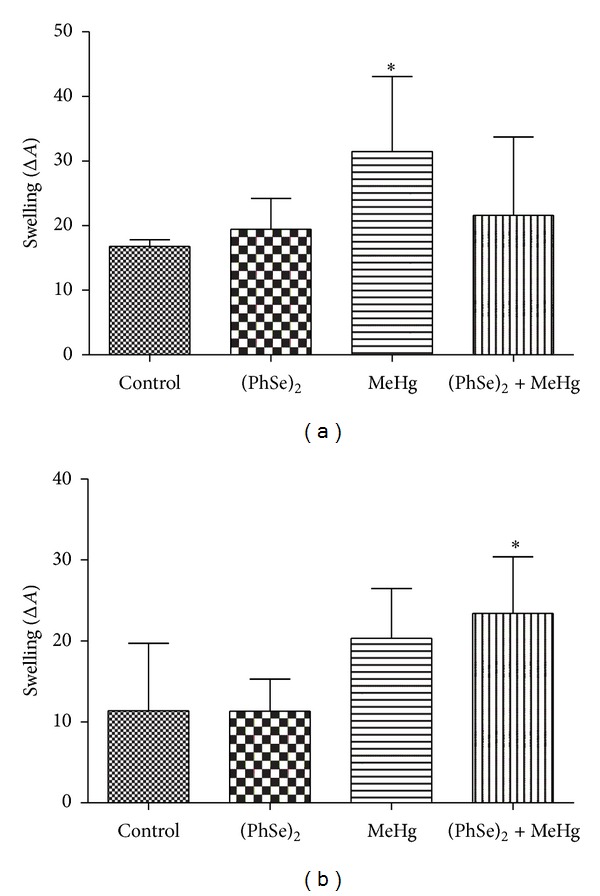
Mitochondrial swelling in liver (a) and brain (b) of rats exposed to MeHg and/or (PhSe)_2_. Data are expressed as mean ± S.D., *n* = 4. (∗) represents *P* < 0.05 as compared to controls by Mann-Whitney test.

Treatment with MeHg significantly increased hepatic mitochondrial swelling when compared to controls (*P* < 0.05, Figure 6(a)). Cotreatment with (PhSe)_2_ partially prevented the MeHg-induced mitochondrial swelling in liver (Figure 6(a)). Similarly, treatment with MeHg showed a trend towards increased mitochondrial swelling in brain (Figure 6(b)). The cotreatment with MeHg and (PhSe)_2_ significantly increased cerebral mitochondrial swelling when compared to controls (*P* < 0.05, Figure 6(b)). Treatment with (PhSe)_2_ alone did not alter the mitochondrial swelling in brain or liver compared to the controls (Figures 6(a) and 6(b)).

#### 3.4.4. Mitochondrial ROS Production

**Figure 7:**
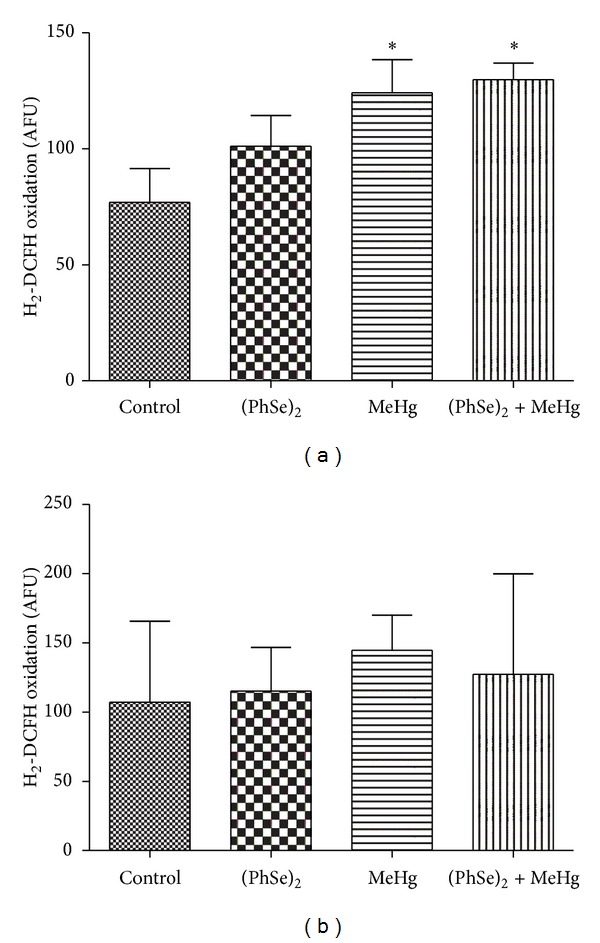
ROS production (H_2_-DCFH oxidation) in liver (a) and brain (b) mitochondria of rats exposed to MeHg and/or (PhSe)_2_. Data are expressed as mean ± S.D., *n* = 4. (∗) represents *P* < 0.05 as compared to controls by Mann-Whitney test.

Mitochondrial ROS production (DCFH oxidation) was significantly increased in livers of rats treated with MeHg or cotreated with MeHg and (PhSe)_2_ (*P* < 0.05, Figure 7(a)). Rats treated with (PhSe)_2_ showed hepatic mitochondrial ROS levels indistinguishable from controls. ROS production in cerebral mitochondria was not affected by any of the treatments (Figure 7(b)).

#### 3.4.5. Mitochondrial ΔΨm

**Figure 8:**
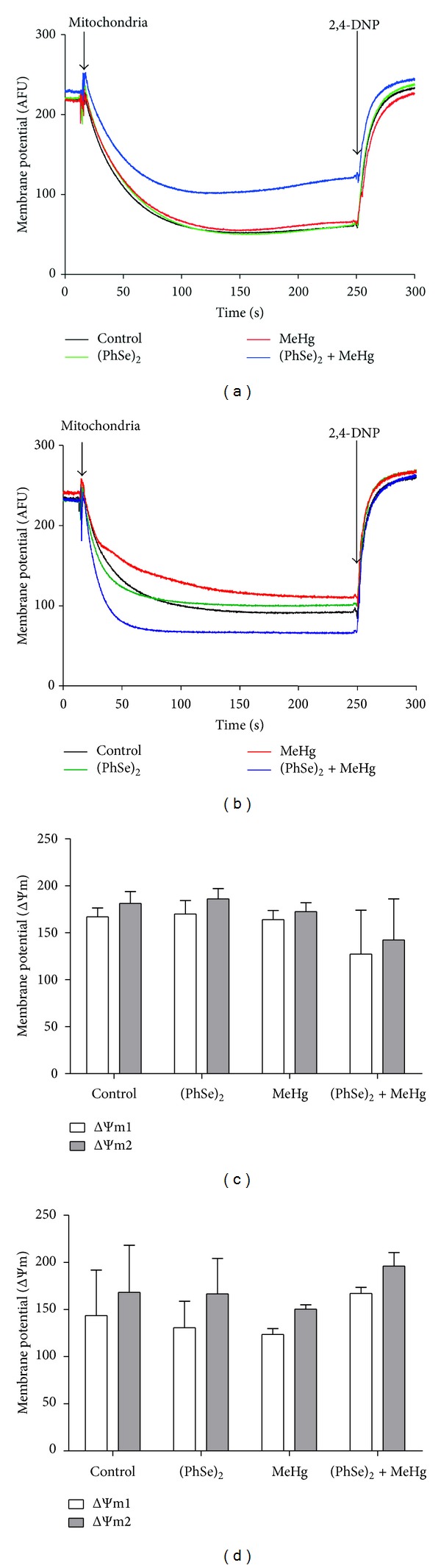
Mitochondrial depolarization in liver (a), (c) and brain (b), (d) of rats exposed to MeHg and/or (PhSe)_2_. Figures (a) and (b) show mitochondrial membrane potential (AFU). Figures (c) and (d) show mitochondrial ΔΨm. ΔΨm1 = delta of fluorescence before (time 0) and after addition of mitochondria (time 150 seconds) and ΔΨm2 = delta of fluorescence before (time 150 seconds) and after addition of 2,4 DNP (time 300 seconds). Data are expressed as mean ± S.D., *n* = 4.

Polarization (ΔΨm) of mitochondria from liver of rats cotreated with MeHg and (PhSe)_2_ showed only a trend towards decreased (Figures 8(a) and 8(c)). Treatment with (PhSe)_2_ and MeHg alone did not cause mitochondrial depolarization in liver of rats (Figures 8(a) and 8(c)). Treatment with (PhSe)_2_ and/or MeHg had no effect on mitochondrial ΔΨm in brain of rats (Figures 8(b) and 8(d)).

### 3.5. Effects of (PhSe)_2_ and MeHg on TrxR Activity

**Figure 9:**
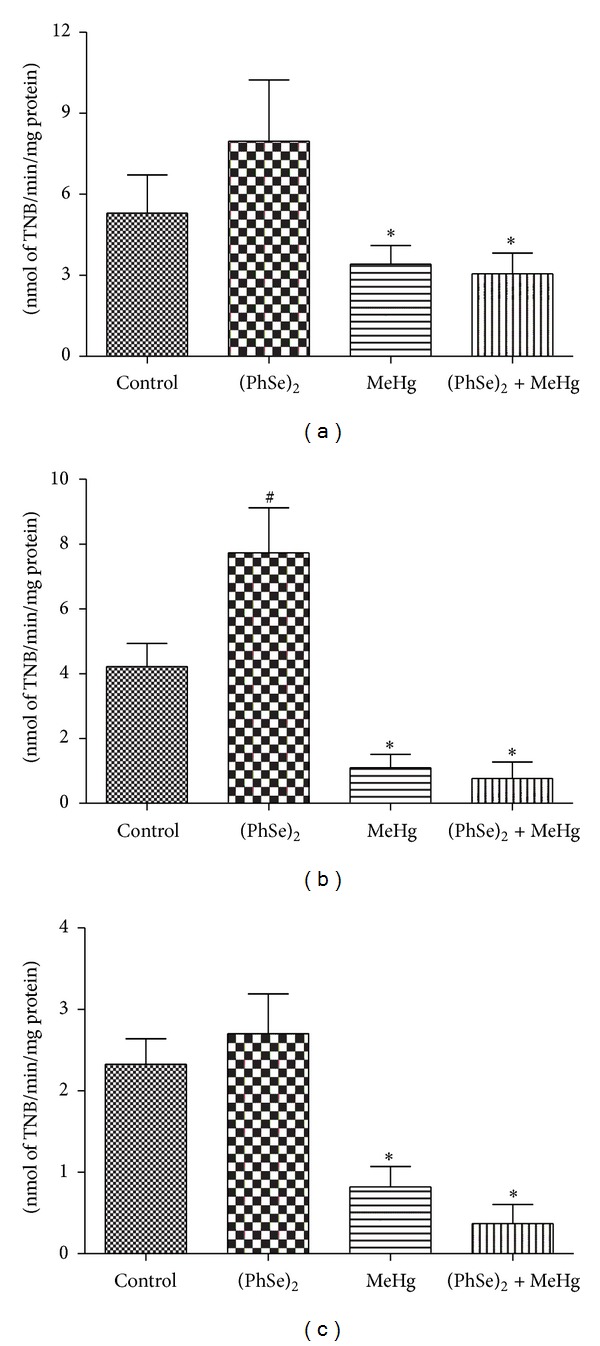
TrxR activity in liver (a), kidney (b), and brain (c) of rats exposed to MeHg and/or (PhSe)_2_. Data are expressed as mean ± S.D., *n* = 4. (∗) represents *P* < 0.05 as compared to controls by Mann-Whitney test. (#) represents *P* < 0.05 as compared to controls by Mann-Whitney test.

MeHg is known to inhibit TrxR activity both *in vitro* and *in vivo* [1, 9, 11]. (PhSe)_2_ treatment significantly increased renal TrxR activities when compared to controls (*P* < 0.05, Figures 9(a) and 9(b)). Hepatic and cerebral TrxR activity showed a trend towards increased in rats treated with (PhSe)_2_ (Figure 9(c)). MeHg treatment also led to significant inhibition of TrxR in liver, kidney, and brain compared to controls (*P* < 0.05, Figure 9). Cotreatment with (PhSe)_2_ failed to significantly attenuate the MeHg-induced inhibition of TrxR activity in the liver, kidney, or brain (Figure 9).

## 4. Discussion

The present study investigated the efficacy of (PhSe)_2_, an organoselenium compound, in attenuating MeHg-induced toxicity in rats. Our results established that MeHg decreased body weight (Figure 1) and induced motor deficits (Figure 3) as well hepatic and cerebral mitochondrial dysfunction (Figures 4(b), 5, 6(a), and 7(a)) and inhibited TrxR activity in liver, brain, and kidney (Figure 9) in the rat. The cotreatment with (PhSe)_2_ and MeHg increased Hg accumulation in the liver and brain (Figure 2). Furthermore, the cotreatment with (PhSe)_2_ protected hepatic and cerebral mitochondrial thiols from depletion by MeHg (Figure 5) but did not prevent hepatic and cerebral mitochondrial dysfunction (Figures 4(b), 6(b), and 7(a)) nor did it reverse the MeHg-induced motor deficits (Figure 3), body-weight loss (Figure 1), and the MeHg-induced inhibition of TrxR activity in liver, brain, and kidney (Figure 9).

Cotreatment with (PhSe)_2_ and MeHg increased Hg deposition in the brain and liver of exposed rats (Figure 2). These results differ from those of de Freitas et al. [21] where (PhSe)_2_ led to a significant reduction in Hg concentrations in brain, liver, and kidney of MeHg-exposed mice. The discrepancies between the 2 studies may be attributed to metabolic differences between the species and the route of administration. The toxicity and pharmacokinetics of MeHg [24] are different in mice and rat which can be explained by the higher binding affinity of rat hemoglobin, containing more cysteinyl residues, for MeHg when compared to the mice hemoglobin [25]. (PhSe)_2_ toxicity and pharmacokinetics differences between mice and rat also exist and could be explained by a faster metabolization of (PhSe)_2_ in mice [26–28]. Notably, herein rats were administered (PhSe)_2_ i.p., whilst in the study by de Freitas et al. [21] (PhSe)_2_ was subcutaneously (s.c.) administered to the mice. Another difference between the two works is in relation to the dose of MeHg: in our study we used a dose 2.5 times higher than in the study of de Freitas et al. (2 mg/Kg). However, the duration of the treatment was shorter in our study, 21 versus 35 days. On the other hand, the dose of (PhSe)_2_ was similar between the two studies. The higher dose of MeHg used in our study may have contributed to the discrepancies since it could generate a more severe toxicity which could not be prevented by (PhSe)_2_. However, we realize that the differences in the pharmacokinetics between rats and mice for the (PhSe)_2_ is the major factor involved in the discrepancies found here [28].

In the study by de Freitas et al. [21] the proposed mechanism for the reduction Hg's organ burden by (PhSe)_2_ was the formation of a selenol/selenolate (PhSeH/PhSe^−^) intermediate, which could interact with MeHg, generating the readily excretable PhSeHgMe complex. One possible explanation for the increase in hepatic and cerebral Hg deposition (Figures 2(a) and 2(b), resp.) by the cotreatment with (PhSe)_2_ observed herein may be the conversion of (PhSe)_2_ to inorganic selenium, which is subsequently metabolized to selenhidric acid (HSe^−^). HSe^−^ could bind to MeHg to form a less soluble complex [45], which can be degraded to HgSe [46, 47]. In addition, Palmer and Parkin [48] showed that organoselenium can also form a complex with mercury. Thus the increase in hepatic and cerebral Hg deposition by the cotreatment with (PhSe)_2_ possibly involves Hg:Se interactions and the formation of a less excretable compound that accumulates in these organs [45]. These results are in agreement with other studies that showed elevated deposition of Hg in key brain regions upon oral Se administration [49, 50]. It has been speculated that the formation of insoluble HgSe salt could reduce the toxicity of MeHg. However, experimental evidence supporting this assumption has yet to be generated. Although the cotreatment with (PhSe)_2_ increased Hg levels in brain and liver, these were accompanied by a partial protection against MeHg-induced mitochondrial dysfunction. We suggest that the formation of an insoluble and inert complex between Hg and Se could decrease the availability of MeHg that could react with important cellular components decreasing its toxicity.

Decreased weight gain and weight loss are prominent and readily observed features of severe MeHg toxicity. In this study, rats treated with MeHg showed body-weight loss (Figure 1). Notably, the most severe effect on weight loss occurred in rats cotreated with (PhSe)_2_ and MeHg (Figure 1). In addition, rats treated with MeHg showed decreased locomotor activity (Figure 3). Cotreatment with (PhSe)_2_ and MeHg increased the severity of motor dysfunction (rotarod test) (Figure 3), likely as a result of increased Hg deposition in the brain (Figure 2(b)). Motor deficits are the most apparent neurological effects following MeHg exposure [51]. *In vivo* studies in rodents point to impairment in intracellular calcium homeostasis, alteration in glutamate homeostasis, and oxidative stress as critical mediators of MeHg-induced neurotoxicity [52]. The overactivation of N-methyl-D-aspartate- (NMDA-) type glutamate receptors increases Ca^2+^ influx into neurons, thereby leading to cell death [53]. Alternatively, Ca^2+^ taken up by mitochondria may stimulate the generation of ROS [54].

Several studies corroborate MeHg's ability to induce mitochondrial dysfunction and ROS generation [14, 18, 55]. The high affinity binding of MeHg to thiol groups inactivates enzymes, including respiratory chain complexes [7, 13, 55], decreasing mitochondrial dehydrogenases activity. Inhibition of these complexes may contribute to mitochondrial swelling and ROS production after MeHg exposure (Figures 6 and 7). However, in brain, the MeHg-induced decrease in mitochondrial dehydrogenases activity (MTT reduction) was not accompanied by an increase in ROS production. These results are corroborated by the fact that MeHg affected total thiols but not nonprotein thiol levels in brain mitochondria. MeHg caused a decrease in the total mitochondrial thiol levels in brain, which is related mainly with protein thiols, and is in agreement with the inhibition of mitochondrial dehydrogenases activity in this tissue. On the other hand, MeHg did not affect nonprotein thiol levels (mainly GSH) in brain mitochondria, which can explain the normal ROS production, since GSH is the main antioxidant in brain.

The cotreatment with (PhSe)_2_ prevented the MeHg-induced mitochondrial total and nonprotein thiol groups depletion in the brain and liver (Figure 4). The efficacy of (PhSe)_2_ in preventing thiol depletion may reside in its ability to form a complex with MeHg, thus effectively reducing MeHg binding to protein and free thiols. Treatment with (PhSe)_2_ also partially protected the liver from mitochondrial MeHg-induced swelling (Figure 6(a)). However, the cotreatment with (PhSe)_2_ failed to reverse the MeHg-induced mitochondrial swelling (Figure 6(b)) and decreased mitochondrial metabolic function (Figure 3(b)) in the brain as well as increased mitochondrial ROS production (Figure 7(a)) in the liver. These results indicate that mechanisms other than the interaction with important free and protein thiols are likely involved in the MeHg-induced mitochondrial dysfunction. Thus, the preferential affinity of MeHg for specific, and as of yet unidentified, mitochondrial protein targets may have a critical role in MeHg's toxicity.

Previous studies have demonstrated that MeHg can directly inhibit TrxR activity both *in vitro* and *in vivo* [1, 9, 11]. Mammalian TrxR is a selenoenzyme containing a unique, catalytically active selenolthiol/selenenylsulfide in the conserved C-terminal sequence (-Gly-Cys-Sec-Gly) [56]. Three mammalian TrxR selenoenzymes have been identified, the cytosolic enzyme TrxR1, the mitochondrial enzyme TrxR2, and a testis-specific enzyme thioredoxin-glutathione reductase (TGR/TrxR3) [56]. Here, we show that MeHg treatment inhibited rat TrxR activity in brain, liver, and kidney (Figure 9). MeHg forms covalent bonds between its Hg moiety and the Se of the selenocysteine of the enzyme, thus directly inhibiting the activity of TrxR [1]. Since TrxR is critical for cellular antioxidant defense system the inhibition of this enzyme likely has a central role in mediating the toxicity of MeHg.

Recently, diphenyl diselenide was demonstrated to be a substrate for cerebral and hepatic rat TrxR, which could account, at least in part, for the antioxidant properties of (PhSe)_2_ [23]. Herein, rats treated solely with (PhSe) showed an increase in the activity of renal TrxR (Figures 9(a) and 9(b), resp.). The formation of selenhidric acid from (PhSe)_2_ could also explain the increase in TrxR activity, since this inorganic form of Se can be converted to selenocysteine and incorporated to selenoenzymes, such as TrxR [45, 57]. Accordingly, Zhang et al. [58] have demonstrated that organoselenium compounds (including diselenide) increase the expression of TrxR in white blood cells lines in culture. The cotreatment with (PhSe)_2_ and MeHg was ineffective in attenuating the inhibition of MeHg-induced TrxR in liver, kidney, and brain (Figure 9). Similarly, studies *in vitro* and *in vivo* have previously corroborated that selenite was able to recover the activity of HgCl_2_-induced TrxR inhibition but not in response to MeHg. The effect of Se (as selenide) was attributed to the displacement of Hg from the active site, giving rise to mercury selenide and regenerating the TrxR selenol [1, 9].

## 5. Conclusions

In conclusion, the results of this study established that (PhSe)_2_ can increase Hg body burden (likely associated with release of inorganic Se from (PhSe)_2_) and MeHg neurotoxicity in rats despite the fact that (PhSe)_2_ blunted the deleterious effects of MeHg on thiol levels. The results presented herein also reinforce the central role of mitochondrial dysfunction in mediating the aberrant effects of MeHg *in vivo*, as well as the role of TrxR as a molecular target for MeHg in the rat. Further research into MeHg-(PhSe)_2_ interactions will be helpful in characterizing the consequences concomitant exposures to these and related compounds.
